# Mind the Gap: House Structure and the Risk of Malaria in Uganda

**DOI:** 10.1371/journal.pone.0117396

**Published:** 2015-01-30

**Authors:** Humphrey Wanzirah, Lucy S. Tusting, Emmanuel Arinaitwe, Agaba Katureebe, Kilama Maxwell, John Rek, Christian Bottomley, Sarah G. Staedke, Moses Kamya, Grant Dorsey, Steve W. Lindsay

**Affiliations:** 1 Infectious Disease Research Collaboration, Mulago Hospital Complex, Kampala, Uganda; 2 Department of Disease Control, London School of Hygiene and Tropical Medicine, London, United Kingdom; 3 Department of Infectious Disease Epidemiology, London School of Hygiene and Tropical Medicine, London, United Kingdom; 4 Department of Clinical Research, London School of Hygiene and Tropical Medicine, London, United Kingdom; 5 Department of Medicine, Makerere University College of Health Science, Kampala, Uganda; 6 Department of Medicine, University of California San Francisco, San Francisco, California, United States of America; 7 School of Biological and Biomedical Sciences, Durham University, Durham, United Kingdom; Institut Pasteur, FRANCE

## Abstract

**Background:**

Good house construction may reduce the risk of malaria by limiting the entry of mosquito vectors. We assessed how house design may affect mosquito house entry and malaria risk in Uganda.

**Methods:**

100 households were enrolled in each of three sub-counties: Walukuba, Jinja district; Kihihi, Kanungu district; and Nagongera, Tororo district. CDC light trap collections of mosquitoes were done monthly in all homes. All children aged six months to ten years (n = 878) were followed prospectively for a total of 24 months to measure parasite prevalence every three months and malaria incidence. Homes were classified as modern (cement, wood or metal walls; and tiled or metal roof; and closed eaves) or traditional (all other homes).

**Results:**

A total of 113,618 female *Anopheles* were collected over 6,765 nights. 6,816 routine blood smears were taken of which 1,061 (15.6%) were malaria parasite positive. 2,582 episodes of uncomplicated malaria were diagnosed after 1,569 person years of follow-up, giving an overall incidence of 1.6 episodes per person year at risk. The human biting rate was lower in modern homes than in traditional homes (adjusted incidence rate ratio (IRR) 0.48, 95% confidence interval (CI) 0.37–0.64, p<0.001). The odds of malaria infection were lower in modern homes across all the sub-counties (adjusted odds ratio 0.44, 95%CI 0.30–0.65, p<0.001), while malaria incidence was lower in modern homes in Kihihi (adjusted IRR 0.61, 95%CI 0.40–0.91, p = 0.02) but not in Walukuba or Nagongera.

**Conclusions:**

House design is likely to explain some of the heterogeneity of malaria transmission in Uganda and represents a promising target for future interventions, even in highly endemic areas.

## Introduction

The population of Africa is expected to double to nearly two billion between 2010 and 2040 and may reach three billion by 2070 [1]. The need to invest in improving and expanding housing options is therefore urgent. Previous studies have demonstrated the importance of house design as a determinant of malaria risk [2–4] and good house construction could prove an important future supplement to long-lasting insecticide-treated nets (LLINs) and indoor residual spraying (IRS) [5,6] [13].

House structure is expected to affect malaria transmission since up to 80–100% of transmission in sub-Saharan Africa occurs indoors [7]. *Anopheles gambiae* s.l., the major African malaria vector, enters houses at night through open eaves, the gap between the top of the wall and the roof [8]. Thus closing the eaves has been observed to be protective against malaria in Ethiopia [9] and The Gambia [10]. [2]Screening external doors and windows is also a simple method to reduce indoor transmission [2,3]. In a recent randomised controlled trial in The Gambia, house screening was associated with a 50% reduction in indoor vector density and a similar 50% reduction in the risk of anaemia in young children [11]. Other potentially protective features include the replacement of thatched roofs with tiled or metal roofs, as observed in Tanzania [12], and the presence of ceilings, as observed in The Gambia [13] and Kenya [14].

**Fig 1:**
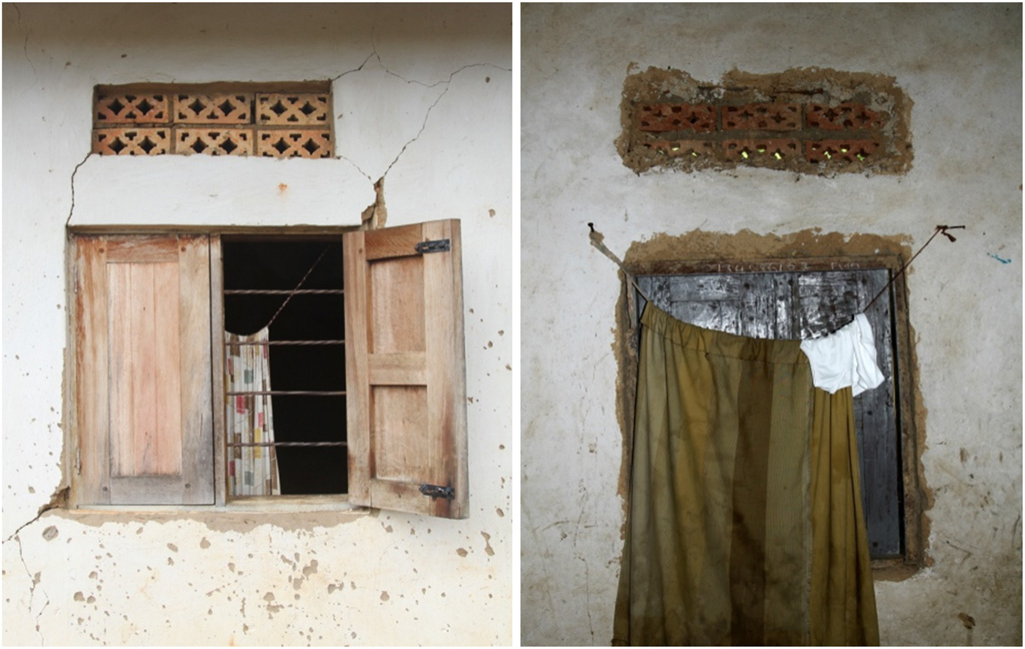
External (left) and internal (right) view of unscreened airbricks.

In Uganda, new homes are typically constructed with metal roofs, brick or concrete walls and closed eaves replacing the traditional thatched roofs, mud walls and open eaves. In urban areas, homes are often built with well-fitted doors and windows to improve security and unscreened airbricks are frequently inserted over doors and windows to cool the interior of these buildings (Fig. 1). Here we investigated whether modern architectural features are associated with reduced house entry by mosquitoes and malaria risk in children at three sites in Uganda with mixed housing and markedly different malaria transmission levels.

## Methods

### Study site

**Fig 2:**
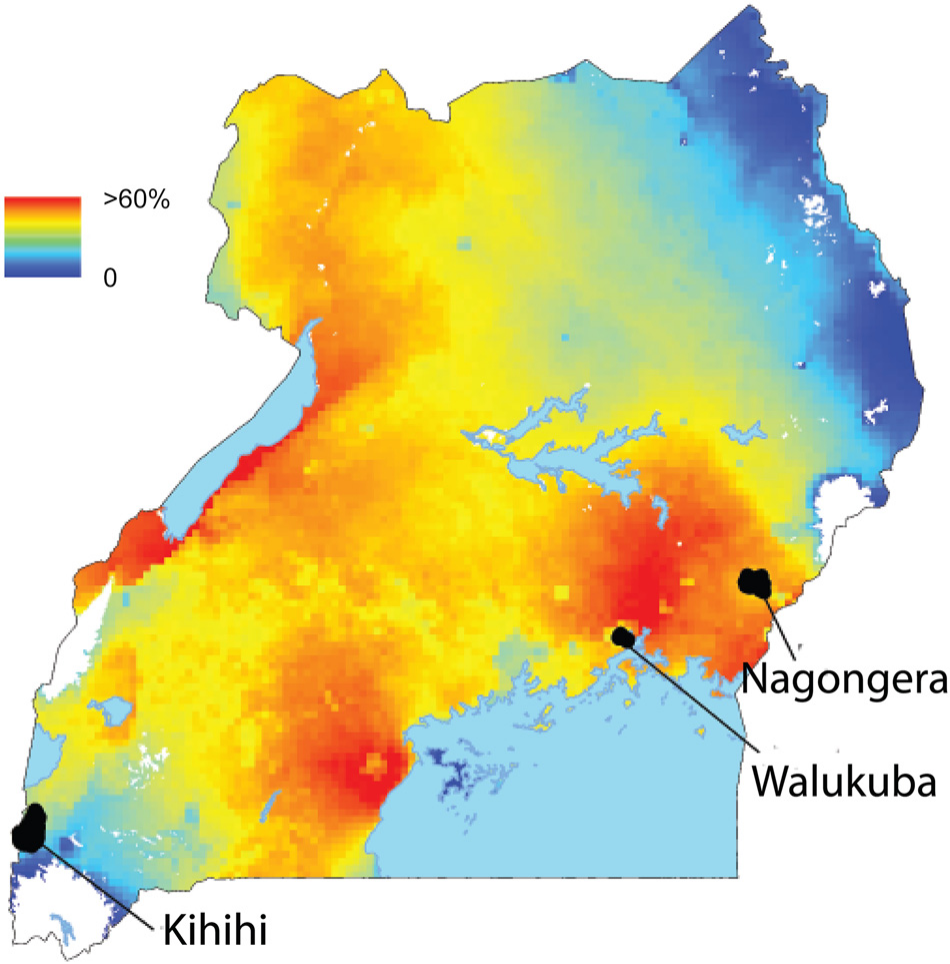
*Plasmodium falciparum* parasite rate (*Pf*PR) and location of study sites in Uganda. The colours represent *Pf*PR in children aged 2–10 years from the Malaria Atlas Project 2010 dataset [16].

The study was carried out in Walukuba sub-county, Jinja district; Kihihi sub-county, Kanungu district and Nagongera sub-country, Tororo district, between August 2011 and September 2013 (Fig. 2). There are two rainy seasons (March to May; August to October). Walukuba (00°26’33.2”N, 33°13’32.3”E) is situated near Lake Victoria. Malaria transmission is low with an estimated annual *Plasmodium falciparum* entomological inoculation rate (a*Pf*EIR) of 2.8 infective bites per person per year [15]. The primary malaria vector species is *An*. *arabiensis* (64%), the remainder being *An*. *gambiae* s.s. (36%) [15].^,^ [20] Kihihi (00°45’03.1”S, 29°42’03.6”E) is a rural setting in the highlands of western Uganda with moderate malaria transmission and an estimated a*Pf*EIR of 32. [18] The primary malaria vector species is *An*. *gambiae* s.s. (99%) [15]. Nagongera (00°46’10.6”N, 34°01’34.1”E) is a rural setting in south-eastern Uganda characterized by savannah grassland, cultivated crops and rocky outcrops. Malaria transmission is extremely high with an estimated a*Pf*EIR of 310. [18] The primary malaria vector species are *An*. *gambiae* s.s. (82%) and *An*. *arabiensis* (19%) [15]. Rainfall patterns are similar in the three study locations. [18]

### Recruitment of study participants

Before the start of the study, a census was conducted in all three sub-counties and a random sample of households selected for screening. From August 2011 to September 2011, children from 100 households randomly selected from the census survey were enrolled into a cohort study and followed for 24 months until September 2013 if they met the following eligibility criteria: (1) aged six months to less than 10 years, (2) resident of the household selected for recruitment, (3) no intention to move out of the sub-county for the next two years, (4) agreement to attend the study clinic for any febrile illness, (5) agreement to avoid antimalarial medications administered elsewhere and (6) provision of written informed consent. Recruitment was dynamic such that children reaching six months of age and meeting the eligibility criteria were enrolled, and children were withdrawn when they reached eleven years of age. The sample size of 300 children for each site was calculated for a separate study comparing temporal changes in malaria incidence from the cohort studies with temporal changes in malaria test positivity rate from health facility based surveillance. The analysis described here is a secondary analysis making use of these data sets. The study data are available in S1 Data.

### Baseline assessment and follow-up of study participants

At enrolment, a baseline clinical evaluation was conducted and study participants were given a LLIN (PermaNet, Vestergaard Frandsen, Switzerland). Parents of participants were requested that their children attend the designated study clinic, open seven days a week, for all healthcare needs. Subjects presenting with a fever or history of fever within the past 24 hours with a positive blood smear were diagnosed with malaria. Episodes of uncomplicated malaria were treated with artemether-lumefantrine and complicated episodes treated with quinine. New episodes of malaria were diagnosed by passive case detection and malaria episodes defined as any treatment for malaria. Routine visits were conducted at the study clinic every three months, with a standard evaluation including a thick blood smear to assess for parasitaemia.

### Microscopy

Thick and thin blood smears were stained with 2% Giemsa and read blind. Blood smears were considered negative when the examination of 100 high power fields did not reveal asexual parasites. All slides were read twice and discrepancies resolved by a third reviewer. In addition, all positive blood smears with a parasite densities ≤20,000/μl based on the field readings were re-read by an expert microscopist based in Kampala and had to be confirmed to be considered positive in the final analyses.

### Entomology

Detailed descriptions of the entomological studies are provided elsewhere [15]. In brief, CDC light trap collections were done monthly in each house for 24 months. Occupants were given a LLIN (PermaNet, Vestergaard Frandsen, Switzerland) and the light trap positioned with the light 1.5m from the floor near the foot of the bed. Collections were made between 19.00h and 07.00h the following morning. Specimens were sorted to species level and counted.

### Household Surveys

Each household was visited at baseline and a questionnaire administered to the head of the household to record data on features of the house (main materials of the wall, roof and floor), which were independently validated by field assistants, together with household demographics and proxy wealth indicators.

### Statistical analysis

Data were collected using a paperless system for the household survey and using standardized case record forms entered into Microsoft Access for follow-up of study participants. Analyses were performed with Stata Version 13 (StataCorp, Texas). Missing data were excluded from the analysis.

### Wealth index and household characteristics

Principal component analysis (PCA) was used to create a wealth index from 10 factors [17]: ownership of (1) mobile telephones, (2) radios, (3) clocks, (4) cupboards, (5) tables, (6) bicycles; (7) number of days that meat was consumed in the past week (<2 versus ≥2 days), (8) difficulty in getting food to eat (sometimes, often or always versus seldom or never), (9) toilet access (no facility, a composting toilet or uncovered pit latrine, versus a covered pit latrine or flush toilet) and (10) main mode of transport to the health facility (walking versus other). Within each study site households were ranked by wealth scores and site-specific tertiles created to provide a categorical measure of socio-economic status. Household characteristics were compared between sites using the chi-square test.

### Entomological and epidemiological outcomes

Main wall material, main roof material and eave type were used to classify homes as either modern (wood, cement or brick walls; and metal or tiled roof; and closed eaves) or traditional (all other homes). Negative binomial regression was used to model the relationship between household risk factors and the number of *Anopheles* caught per house by light trap catches, with the number of sampling nights included as an offset term in the model. The odds of malaria infection at the time of each routine clinic visit was modelled using logistic regression and negative binomial regression used to model the number of malaria cases per child. Robust standard errors were used to adjust for clustering due to household in both models. We estimated an incidence rate ratio (IRR) for the association between house type and human biting rate (HBR) adjusted for household wealth; and an odds ratio and incidence rate ratio for the association between house type and malaria adjusted for age, gender and household wealth. The associations were analysed separately for each study site and Wald tests were used to test for effect modification by study site.

### Ethics

Written informed consent was obtained in the appropriate language from guardians for the participation of their child and from an adult household member for the light trap catches and household surveys. Approval from local leaders was obtained before beginning activities. Ethics approval was provided by the Uganda National Council for Science and Technology; Makerere University School of Medicine Research and Ethics Committee; University of California, San Francisco Committee for Human Research; and the London School of Hygiene and Tropical Medicine Ethics Committee.

## Results

### Study population

**Fig 3:**
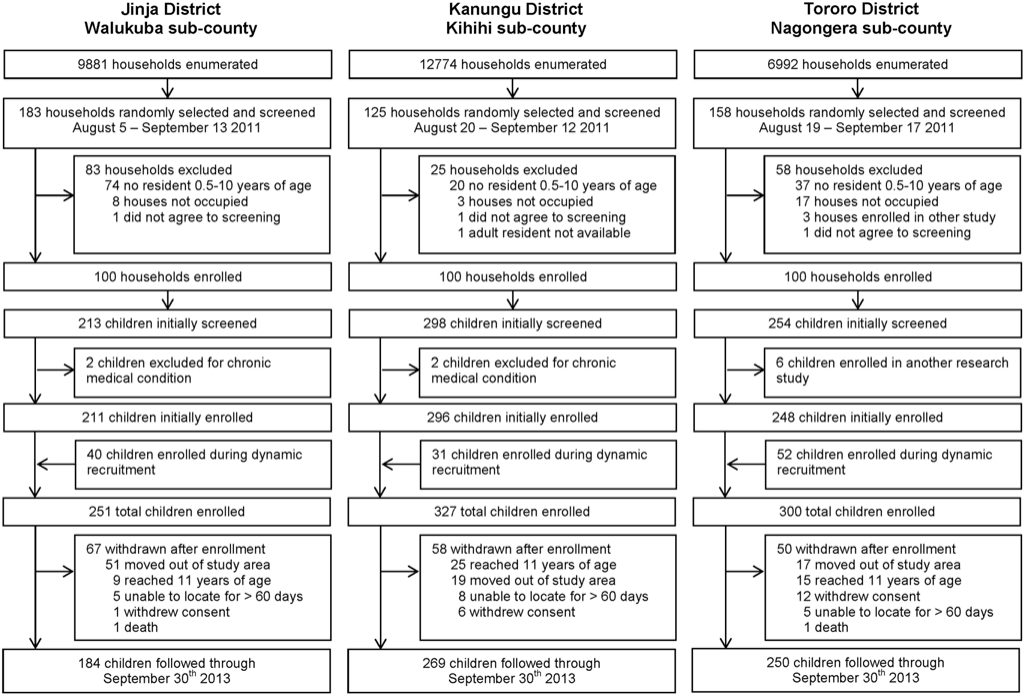
Study profile.

In total 878 children were enrolled; 251 in Walukuba, 327 in Kihihi and 300 in Nagongera (Fig. 3; Table 1). The mean age of participants during follow-up was five years and 428 (48.8%) were female. Overall, 103 of 300 (34.3%) homes were classified as modern (with cement, wood or metal walls, tiled or metal roofs and closed eaves), 114 (38.0%) had unscreened airbricks and 21 (7.0%) had screened airbricks. Homes in peri-urban Walukuba were generally of better quality than those in rural Kihihi and Nagongera (Table 1).

**Table 1:** Characteristics of study participants and households

Characteristic		All sites	Individual study sites			
			Walukuba	Kihihi	Nagongera	P
Individual study participant level data						
Number of children		878	251	327	300	-
Mean age in years during follow up (95% CI^a^)		5.4 (5.2–5.6)	5.0 (4.7–5.4)	5.6 (5.3–5.9)	5.4 (5.1–5.8)	0.06
Female participants (%)		428 (48.8%)	127 (50.6%)	165 (50.5%)	136 (45.3%)	0.35
Individual household level data						
Number of households		300	100	100	100	
Wealth index, stratified by study site (%)	Poorest tertile	34.7	34	36	34	
	Medium tertile	34.3	35	35	33	
	Highest tertile	31.0	31	29	33	0.98
Main floor material (%)	Earth, sand, dung or stones	69.7	48	77	84	
	Wood, bricks or cement	30.3	52	23	16	<0.001
Main roof material (%)	Thatched	17.7	2	13	38	
	Tiles or metal	82.3	98	87	62	<0.001
Main wall material (%)	Mud	60.7	35	70	77	
	Cement, wood or metal	39.3	65	30	23	<0.001
Eaves (%)	Open	33.7	28	25	48	
	Closed	66.3	72	75	52	0.001
Airbricks (%)	None	55.0	68	27	70	
	Unscreened	38.0	14	72	28	
	Screened	7.0	18	1	2	<0.001
House type (%)	Traditional	65.7	49	71	77	
	Modern^b^	34.3	51	29	23	<0.001

^a^Confidence interval.

^b^Cement, wood or metal wall; tiled or metal roof and closed eaves.

### Wealth index

The first principal component explained 21.5% of the overall variability in the asset variables. The weight assigned to each variable was: radio ownership (0.43), table ownership (0.41), cupboard ownership (0.39), mobile ownership (0.34); frequency of problems satisfying food needs (0.33), toilet access (0.33), clock ownership (0.32), bicycle ownership (0.18), main mode of transport to health facility (0.14), and meat consumption (0.12).

### Human biting rate

**Fig 4:**
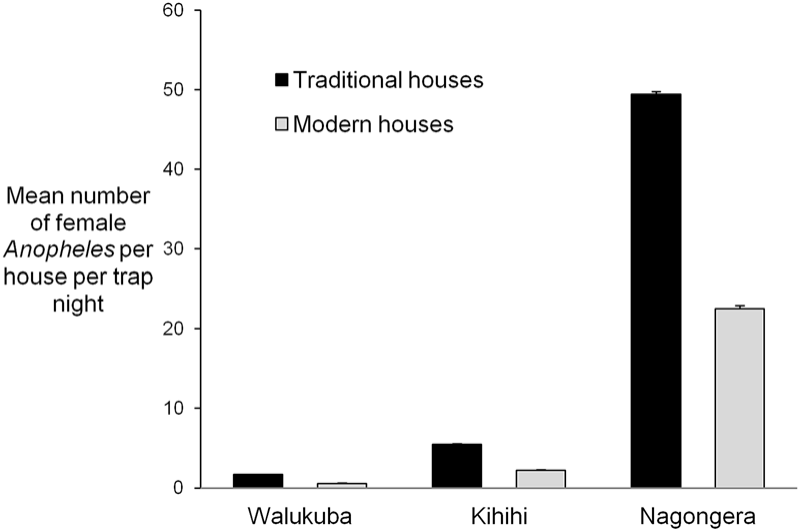
Mean human biting rate (Anopheles spp) in houses classified as modern (cement, wood or metal wall; tiled or metal roof and closed eaves) and traditional (all other houses) at three sites in Uganda. Error bars represent upper 95% confidence intervals.

113,618 adult female *Anopheles* were collected over 6,765 nights of collection. Data were missing for one household. Overall, HBR was highest in Nagongera (43.3 adult female *Anopheles* per house per night) and lower in Kihihi (4.6) and Walukuba (1.1). In Kihihi and Nagongera, HBR was lower in homes with tiled or metal roofs and homes with cement, wood or metal walls (Table 2). At all sites HBR was lower in houses with closed eaves than houses with open eaves, and in houses with screened or unscreened airbricks compared to houses with no airbricks. There was no evidence that the association between house type and HBR varied with site. Controlling for site and household wealth, the human biting rate was 52% lower in modern homes compared with traditional homes (incidence rate ratio (IRR) 0.48, 95% confidence interval (CI) 0.37–0.64, p<0.001, Fig. 4).

**Table 2:** Association between household characteristics and the human biting rate at three sites in Uganda.

Characteristic		Walukuba			Kihihi			Nagongera		
		HBR^a^ (Total collection nights)	IRR (95% CI)^b^	p	HBR (Total collection nights)	IRR (95% CI)^b^	p	HBR (Total collection nights)	IRR (95% CI)^b^	p
Wealth index, stratified by study site	1^st^ tertile	1.97 (692)	1	-	7.92 (830)	1	-	49.24 (787)	1	-
	2^nd^ tertile	0.75 (777)	0.30 (0.18–0.52)	<0.001	2.58 (795)	0.33 (0.20–0.56)	<0.001	40.57 (785)	0.79 (0.58–1.07)	0.13
	3^rd^ tertile	0.58 (715)	0.25 (0.14–0.43)	<0.001	2.78 (627)	0.38 (0.22–0.66)	0.001	40.02 (757)	0.76 (0.56–1.04)	0.09
Main floor material	Earth, sand, dung or stones	1.76 (1010)	1	-	5.32 (1778)	1	-	46.93 (1966)	1	-
	Wood, bricks or cement	0.49 (1174)	0.25 (0.16–0.40)	<0.001	1.93 (474)	0.38 (0.22–0.65)	0.001	23.74 (363)	0.49 (0.35–0.68)	<0.001
Main roof material	Thatched	1.17 (48)	1	-	8.95 (307)	1	-	54.38 (872)	1	-
	Tiles or metal	1.08 (2136)	1.07 (0.18–6.36)	0.94	3.92 (1945)	0.43 (0.22–0.86)	0.02	36.70 (1457)	0.65 (0.50–0.83)	0.001
Main wall material	Mud	1.65 (698)	1	-	5.52 (1636)	1	-	49.42 (1800)	1	-
	Cement, wood or metal	0.81 (1486)	0.63 (0.37–1.06)	0.08	2.18 (616)	0.40 (0.24–0.66)	<0.001	22.54 (529)	0.45 (0.34–0.58)	<0.001
Eaves	Open	1.73 (616)	1	-	7.63 (579)	1	-	53.86 (1109)	1	-
	Closed	0.83 (1568)	0.39 (0.23–0.67)	0.001	3.56 (1673)	0.45 (0.27–0.77)	0.004	33.74 (1220)	0.60 (0.48–0.77)	<0.001
Airbricks	None	1.39 (1456)	1	-	7.50 (608)	1	-	48.48 (1615)	1	-
	Unscreened	0.56 (321)	0.34 (0.17–0.68)	0.002	3.58 (1621)	0.48 (0.29–0.80)	0.005	33.44 (667)	0.68 (0.52–0.89)	0.004
	Screened	0.39 (407)	0.26 (0.14–0.49)	<0.001	0.43 (23)	0.06 (0.01–0.65)	0.02	6.21 (47)	0.13 (0.05–0.30)	<0.001
House type^c^	Traditional	1.68 (1010)	1	-	5.46 (1659)	1	-	49.42 (1800)	1	-
	Modern^d^	0.57 (1174)	0.49 (0.28–0.85)	0.01	2.21 (593)	0.54 (0.30–0.98)	0.04	22.54 (529)	0.45 (0.34–0.61)	<0.001

^**a**^HBR: Human biting rate, adult female anopheles collected per house per night (total adult female anophelines caught / total nights of collection).

^b^IRR: Incidence rate ratio; CI: Confidence interval.

^c^IRR adjusted for household wealth.

^d^Cement, wood or metal wall; tiled or metal roof and closed eaves.

### Malaria infection

6,816 routine blood smears were taken of which 1,061 (15.6%) were positive. All children contributed at least one routine blood smear. *Pf*PR was highest in Nagongera (28.7%) and lower in Kihihi (9.4%) and Walukuba (7.4%). The association between house type and odds of malaria infection varied by site (p<0.001). Controlling for age, gender and household wealth, the odds of malaria infection were lower in children living in modern homes than in traditional homes in Walukuba (Odds Ratio (OR) 0.35, 95%CI 0.13–0.92, p = 0.03), Kihihi (OR = 0.27, 95%CI 0.10–0.71, p = 0.008) and Nagongera (OR 0.59, 95%CI 0.38–0.90, p = 0.01) (Table 3). Overall, controlling for age, gender, site and household wealth, the odds of malaria infection were 56% lower in children living in modern homes (OR 0.44, 95%CI 0.30–0.65, p<0.001).

**Table 3:** Risk factors for malaria infection in children aged 6 months to 10 years at three sites in Uganda.

Characteristic		Walukuba			Kihihi			Nagongera		
		PR^a^ (Total blood smears)	OR (95% CI)^b^	p	PR (Total blood smears)	OR (95% CI)	p	PR (Total blood smears)	OR (95% CI)	p
Age at time of blood smear	6m to <3 years	5.3 (455)	1	-	6.0 (598)	1	-	18.5 (491)	1	-
	3 to <5 years	8.4 (441)	1.64 (0.88–3.06)	0.12	8.8 (543)	1.51 (0.88–2.61)	0.14	27.0 (514)	1.63 (1.13–2.35)	0.01
	5 to <11 years	8.0 (929)	1.55 (0.88–2.75)	0.13	10.9 (1470)	1.91 (1.17–3.12)	0.01	32.9 (1375)	2.15 (1.55–2.99)	<0.001
Gender	Female	6.4 (909)	1	-	7.9 (1274)	1	-	27.4 (1077)	1	-
	Male	8.4 (916)	1.35 (0.89–2.04)	0.16	10.8 (1337)	1.42 (0.97–2.08)	0.08	29.7 (1303)	1.12 (0.88–1.43)	0.37
Wealth index, stratified by study site	1^st^ tertile	8.4 (526)	1	-	13.5 (1001)	1	-	33.3 (771)	1	-
	2^nd^ tertile	7.0 (616)	0.82 (0.38–1.78)	0.62	7.8 (883)	0.54 (0.28–1.06)	0.07	26.6 (888)	0.72 (0.50–1.04)	0.08
	3^rd^ tertile	7.0 (683)	0.83 (0.31–2.18)	0.70	5.5 (727)	0.37 (0.20–0.71)	0.003	26.2 (721)	0.71 (0.47–1.07)	0.10
Main floor material	Earth, sand, dung or stones	10.0 (869)	1	-	10.6 (2130)	1	-	30.0 (2113)	1	-
	Wood, bricks or cement	5.0 (956)	0.48 (0.23–0.99)	0.05	3.7 (481)	0.33 (0.13–0.80)	0.01	18.4 (267)	0.53 (0.36–0.76)	0.001
Main roof material	Thatched	7.4 (27)	1	-	16.6 (314)	1	-	24.5 (918)	1	-
	Tiles or metal	7.4 (1798)	1.00 (0.34–2.92)	0.99	8.4 (2297)	0.46 (0.23–0.92)	0.03	31.3 (1462)	1.40 (1.05–1.87)	0.02
Main wall material	Mud	9.3 (589)	1	-	11.6 (1982)	1	-	30.5 (1977)	1	-
	Cement, wood or metal	6.5 (1236)	0.67 (0.33–1.37)	0.27	2.4 (629)	0.19 (0.07–0.49)	0.001	19.6 (403)	0.56 (0.37–0.84)	0.01
Eaves	Open	10.8 (518)	1	-	17.3 (648)	1	-	27.7 (1161)	1	-
	Closed	6.0 (1307)	0.53 (0.23–1.21)	0.13	6.7 (1963)	0.35 (0.20–0.60)	<0.001	29.5 (1219)	1.09 (0.80–1.49)	0.58
Airbricks	Unscreened	6.3 (256)	1	-	7.3 (1924)	1	-	27.3 (634)	1	-
	Screened	3.6 (331)	0.56 (0.13–2.54)	0.46	0 (10)	-	-	11.1 (18)	0.33 (0.07–1.66)	0.18
	None	8.6 (1238)	1.42 (0.43–4.67)	0.57	15.2 (677)	2.27 (1.30–3.98)	0.004	29.3 (1728)	1.11 (0.75–1.63)	0.61
House type^c^	Traditional	10.7 (857)	1	-	11.4 (2017)	1	-	30.5 (1977)	1	-
	Modern^d^	4.4 (968)	0.35 (0.13–0.92)	0.03	2.5 (594)	0.27 (0.10–0.71)	0.008	19.6 (403)	0.59 (0.38–0.90)	0.01

^**a**^PR: Parasite rate (total positive blood smears / total blood smears); N: total blood smears.

^b^OR: Odds ratio; CI: Confidence interval.

^c^OR adjusted for age at the time of the blood smear, gender and household wealth.

^d^Cement, wood or metal wall; tiled or metal roof and closed eaves.

### Incidence of clinical malaria

2,582 episodes of uncomplicated malaria were diagnosed after 1,569 person years of follow-up, yielding an overall incidence of 1.6 episodes per person year at risk (PPY). Five participants were withdrawn immediately after screening and did not contribute person years at risk. Incidence was highest in Nagongera (2.8 episodes PPY) and lower in Kihihi (1.4) and Walukuba (0.4). The association between house type and malaria incidence varied by site (p = <0.001). Controlling for age, gender and household wealth, malaria incidence was 39% lower in children living in modern homes in Kihihi (IRR 0.61, 95%CI 0.40–0.91, p = 0.02) but not in Walukuba or Nagongera (Table 4).

**Table 4:** Risk factors for clinical malaria in children aged 6 months to 10 years at three sites in Uganda.

Characteristic		Walukuba			Kihihi			Nagongera		
		Malaria incidence^a^ (total person years)	IRR (95% CI)^b^	p	Malaria incidence (total person years)	IRR (95% CI)	p	Malariaincidence (total person years)	IRR (95% CI)	p
Mean age during follow–up	6m to <3 years	0.40 (106.0)	1	-	1.58 (139.4)	1	-	4.27 (110.8)	1	-
	3 to <5 years	0.62 (93.6)	1.59 (0.92–2.73)	0.10	1.77 (104.0)	1.10 (0.77–1.55)	0.61	3.64 (110.1)	0.86 (0.73–1.02)	0.08
	5 to <11 years	0.37 (223.1)	0.90 (0.61–1.34)	0.62	1.28 (352.9)	0.79 (0.60–1.03)	0.08	2.04 (328.8)	0.48 (0.40–0.58)	<0.001
Gender	Female	0.41 (209.8)	1	-	1.24 (290.4)	1	-	2.51 (248.2)	1	-
	Male	0.45 (212.8)	1.13 (0.73–1.74)	0.59	1.62 (305.9)	1.30 (1.01–1.68)	0.04	3.06 (301.5)	1.24 (1.03–1.49)	0.02
Wealth index, stratified by study site	1^st^ tertile	0.63 (120.8)	1	-	1.85 (227.5)	1	-	3.03 (178.0)	1	-
	2^nd^ tertile	0.40 (142.4)	0.63 (0.31–1.28)	0.20	1.54 (203.7)	0.84 (0.60–1.19)	0.33	3.06 (204.3)	1.00 (0.77–1.28)	0.98
	3^rd^ tertile	0.31 (159.4)	0.49 (0.27–0.87)	0.02	0.73 (165.1)	0.39 (0.26–0.60)	<0.001	2.27 (167.4)	0.72 (0.53–0.98)	0.04
Main floor material	Earth, sand, dung or stones	0.52 (201.0)	1	-	1.55 (486.8)	1	-	2.88 (487.3)	1	-
	Wood, bricks or cement	0.35 (221.6)	0.68 (0.40–1.17)	0.17	0.91 (109.5)	0.58 (0.39–0.86)	0.01	2.26 (62.4)	0.78 (0.49–1.24)	0.30
Main roof material	Thatched	0.63 (6.3)	1	-	2.63 (72.0)	1	-	3.14 (211.5)	1	-
	Tiles or metal	0.43 (416.3)	0.70 (0.49–1.02)	0.06	1.27 (524.4)	0.49 (0.31–0.76)	0.002	2.61 (338.1)	0.82 (0.66–1.02)	0.07
Main wall material	Mud	0.56 (135.0)	1	-	1.66 (453.6)	1	-	2.89 (455.6)	1	-
	Cement, wood or metal	0.37 (287.7)	0.65 (0.36–1.17)	0.15	0.71 (142.7)	0.42 (0.28–0.64)	<0.001	2.44 (94.1)	0.84 (0.57–1.24)	0.39
Eaves	Open	0.49 (120.5)	1	-	2.10 (148.9)	1	-	2.98 (267.9)	1	-
	Closed	0.41 (302.2)	0.81 (0.43–1.51)	0.50	1.21 (447.4)	0.59 (0.40–0.86)	0.01	2.65 (281.7)	0.89 (0.72–1.11)	0.30
Airbricks	Unscreened	0.43 (59.9)	1	-	1.15 (439.1)	1	-	2.66 (147.4)	1	-
	Screened	0.29 (76.7)	0.63 (0.23–1.77)	0.38	0.00 (2.1)	-	-	1.45 (4.1)	0.55 (0.21–1.43)	0.22
	None	0.47 (286.1)	1.03 (0.50–2.12)	0.93	2.26 (155.2)	1.92 (1.35–2.73)	<0.001	2.88 (398.1)	1.09 (0.83–1.43)	0.54
House type^c^	Traditional	0.53 (197.2)	1	-	1.63 (461.7)	1	-	2.89 (455.6)	1	-
	Modern^d^	0.35 (225.4)	0.80 (0.46–1.39)	0.43	0.74 (134.6)	0.61 (0.40–0.91)	0.02	2.44 (94.1)	0.90 (0.63–1.28)	0.55

^**a**^Malaria incidence per person years (new malaria episodes/person years of observation)

^b^IRR: Incidence rate ratio; CI: Confidence interval.

^c^IRR adjusted for mean age during follow up, gender and household wealth.

^d^Cement, wood or metal wall; tiled or metal roof and closed eaves.

## Discussion

We investigated the association between house construction and malaria at three sites in Uganda: peri-urban Walukuba with low malaria transmission, rural Kihihi with moderate transmission and rural Nagongera with high transmission. Modern homes were associated with a 52% reduction in HBR after controlling for site and household wealth. Similarly, the odds of malaria infection were 56% lower in children living in modern homes than those living in traditional homes, after controlling for age, gender, site and household wealth. These results show that reducing vector biting rates by half is associated with a similar proportional reduction in malaria infection. A similar result was found in a randomised controlled trial of house screening in The Gambia which showed that house screening reduced malaria transmission by half, with a similar reduction in the risk of malaria anaemia [11].

Our findings suggest that good house construction may help protect against malaria in Uganda by reducing house entry by vectors. HBR was highest in homes with mud walls, thatched roofs and open eaves, consistent with the house-entering behaviour of *An*. *gambiae*. This vector follows human odour plumes until it reaches an external house wall, flies upwards and, funnelled by the inclined roof, enters the house through open eaves [8,10]. Homes with earth, sand, dung or stone flooring were also crudely associated with a higher HBR and odds of malaria infection than homes with wood, brick or cement floors, perhaps because they are more likely to contain moist, odorous convection currents. Surprisingly, HBR was higher in homes with no airbricks than homes with unscreened airbricks, most likely because houses with airbricks are typically those built in a more modern style, with fewer overall entry points. Screening air bricks with fly mesh to further reduce indoor mosquito density could be further investigated as a cheap and simple additional intervention in well-built homes.

Heterogeneity in malaria transmission at small spatial scales is not only driven by environmental factors such as proximity to larval habitats, but also wealth inequalities [18]. The odds of malaria infection are approximately doubled in the poorest children compared to the wealthiest children within a community [19]. While the exact mechanism for this is unknown, wealthier homes may have improved ownership and use of LLINs [20], [9] better access to chemoprophylaxis and treatment [21], better nutrition and improved [10]treatment-seeking behaviour and health expenditure [22], [27] in addition to better housing [23]. Our findings are consistent with the hypothesis that housing may contribute to socioeconomic inequalities in malaria risk.

We also observed that the association between house type and malaria prevalence and incidence varied by site. This effect modification might be explained by differences in the average quality of homes between sites and a community-level protective effect of good housing. Malaria transmission is generally lower in urban than rural Africa [24] because the built-up environment is less conducive to breeding by *An*. *gambiae*, urban populations generally have better access to prophylaxis and treatment and individual exposure to infectious bites declines with increasing population density [24,25]. [27] The lower HBR and associated burden of malaria observed in peri-urban Walukuba, compared to rural Kihihi and Nagongera, is consistent with the quality of homes in Walukuba being generally higher than the other sites.

Reducing the number of entry points into a house is not a panacea. Most obviously, interventions built into the home do not protect against outdoor transmission [26]. We observed an association between house type and malaria incidence only in Kihihi, where 99% of transmission is by *An*. *gambiae s*.*s*., a highly endophagic vector. In contrast, no association was observed in Walukuba, where 64% of malaria vectors are the less endophagic *An*. *arabiensis*, nor in Nagongera, where 19% vectors are *An*. *arabiensis*. Screening interventions also may not be as effective against culicine mosquitoes as *An*. *gambiae*, reducing their potential appeal to homeowners. Moreover, restricting air flow in homes may increase the internal temperature and the risk from respiratory diseases, especially if wood is burned indoors [27]. However, houses with metal roofs, closed eaves, tightly fitting doors and windows and air bricks are considered desirable, and are being built today on a massive scale [6]. Screening should be further evaluated as a potentially simple and cheap means to reduce malaria risk.

Our findings may also not be generalisible to other countries with different house styles and vector ecology. Furthermore, the observed association between house type and malaria risk is not evidence of causality. Indeed, since the direct and indirect costs of malaria can contribute to poverty within a household [28], especially in low-income settings lacking social security systems, a high malaria burden could plausibly be associated with poorer housing, through its effect on household disposable income and the affordability of building materials. Yet the elevated HBR observed in homes with mud walls, thatched roofs and open eaves is consistent with a direct causal link between house quality and malaria transmission. Household welfare is important to quantify accurately, since house construction is related to wealth, however the ranking of households in our wealth index will have been affected by the indicators selected into the PCA [29]. House design was also assessed only at baseline, without measurement of incremental improvements subsequently accrued. Nonetheless our observations are consistent with an increasing body of work that demonstrates that house features affect mosquito-house entry [4–6].

We provide evidence that house structure may explain some of the often marked heterogeneity of transmission in Uganda. Improving house design should be further evaluated as a potential malaria control intervention in sub-Saharan Africa, even in areas of very high transmission.

## Supporting Information

S1 Data Data files for the manuscript .
(ZIP)
